# Supplementary figures and images for: The Route of Vaccine Administration Determines Whether Blood Neutrophils Undergo Long-Term Phenotypic Modifications
Source: Front Immunol. 2022 Jan 4;12:784813. doi: 10.3389/fimmu.2021.784813 (PMC8764446; doi:10.3389/fimmu.2021.784813)

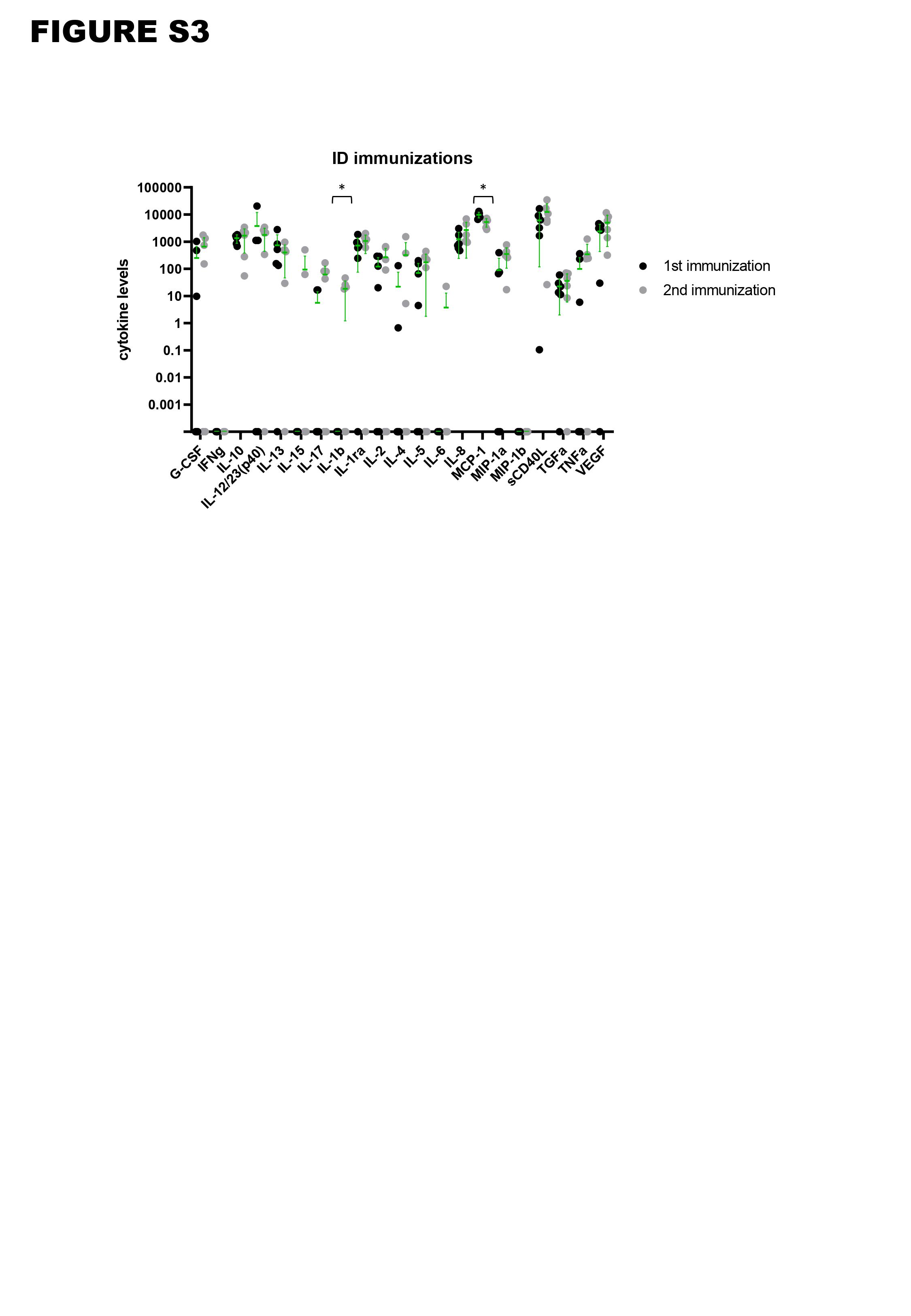

Supplement: Supplementary Figure S1 — Control samples. The same fixed and frozen control samples were stained and acquired with the samples from the vaccinated animals after ex vivo restimulation with a mixture of TLR ligands. (A) Gating strategy to define the CD66high HLA-DR-, CD66-/mid HLA-DR-, and CD66-/mid HLADR+ cell populations. The non-stimulated control sample for the first staining/acquisition session is shown. (B) Comparison of the staining profiles using overlaid histograms. [file Image_1.jpeg]

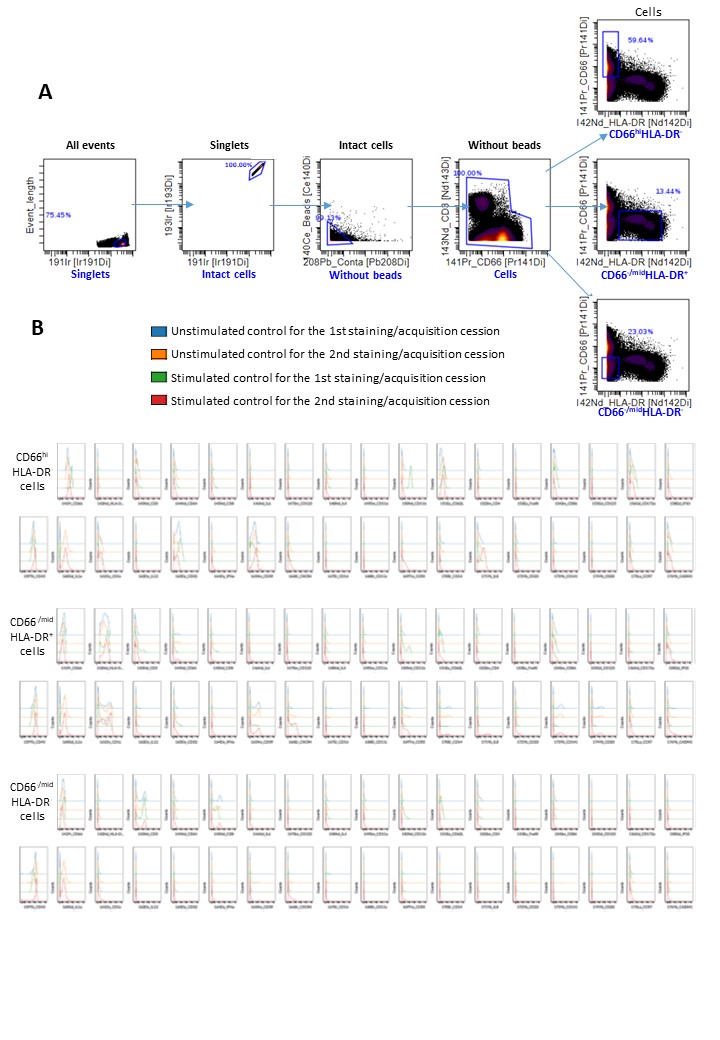

Supplement: Supplementary Figure S3 — Cytokine and chemokine expression profiles in blood after one or two ID MVA immunizations. The plasma concentration of 22 cytokines was measured before, 1 day, 7 days, and 14 days after one or two doses of MVA injected ID (n = 6). Areas under the curve (AUC) from H0 to D14 post-immunization were calculated to represent an approximation of exposure over time, in pg×ml-1×day. The mean AUC of each cytokine after the first and second MVA ID injection is shown as a graph. Zero values are arbitrarily represented on the X axis. Statistically significant differences (p < 0.05, Wilcoxon tests) between the 1st and 2nd injection are indicated by an asterisk (*). [file Image_3.jpeg]
