# Supplementary material for: The Route of Vaccine Administration Determines Whether Blood Neutrophils Undergo Long-Term Phenotypic Modifications
Source: Front Immunol. 2022 Jan 4;12:784813. doi: 10.3389/fimmu.2021.784813 (PMC8764446; doi:10.3389/fimmu.2021.784813)
Supplement: Supplementary Table S1 — Antibody panel for mass cytometry. [file Table_1.docx]

| **Metal** | **Marker** | **Clone** | **Supplier** | **Catalog number** | **µg/3 millions leukocytes** | **Surface** | **Intra-cellular** |
| --- | --- | --- | --- | --- | --- | --- | --- |
| 141Pr | CD66abce | TET2 | Miltenyi | 120-014-229 | 0.3 | ● |  |
| 142Nd | HLA-DR | L243 | Ozyme | 307651 | 0.2 | ● |  |
| 143Nd | CD3 | SP34.2 | BD | 551916 | 2.5 | ● |  |
| 144Nd | CD64 | 10.1 | Miltenyi | 120-014-229 | 1.5 | ● |  |
| 145Nd | CD8 | RPAT8 | BD | 557084 | 1 | ● |  |
| 146Nd | IL-6 | MQ2.13A5 | BD | 554543 | 2 |  | ● |
| 147Sm | CD123 | 7G3 | BD | 554527 | 1 | ● |  |
| 148Nd | IL-4 | 8D48 | BD | 554515 | 2 |  | ● |
| 149Sm | CD11a | HI111 | Miltenyi | 120-014-229 | 2 | ● |  |
| 150Nd | CD11b | ICRF144 | BD | 555386 | 0.5 | ● |  |
| 151Eu | CD62L | SK11 | Miltenyi | 120-014-229 | 2 | ● |  |
| 152Sm | CD4 | L200 | BD | 550625 | 2 | ● |  |
| 153Eu | FcεRI | AER37 (CRA1) | eBioscience  (ThermoFisher Scientific) | 14-5899-82 | 1.5 | ● |  |
| 154Sm | CD86 | IT2.2 | BD | 555663 | 1 | ● |  |
| 155Gd | CD125 | A14 | BD | 624084 | 0.5 | ● |  |
| 156Gd | CD172a | REA144 | Miltenyi | 120-014-229 | 1.5 | ● |  |
| 158Gd | IP-10 | 6D4 | Miltenyi | 120-014-229 | 1 |  | ● |
| 159Tb | CD45 | D058-1283 | BD | 552566 | 0.2 | ● |  |
| 160Gd | IL-1α | 364/3B3 | Miltenyi | 120-014-229 | 2 |  | ● |
| 161Dy | CD1c | AF5910 | R&D systems (Biotechne) | AF5910 | 0.5 | ● |  |
| 162Dy | IL-12 | C8.6 | Miltenyi | 120-014-229 | 1 |  | ● |
| 163Dy | CD32 | FLI8.26 | BD | 555447 | 0.5 | ● |  |
| 164Dy | IFNα | LT27/295 | Miltenyi | 120-014-229 | 0.5 |  | ● |
| 165Ho | CD39 | eBioA1 | Biolegend | 328002 | 1 | ● |  |
| 166Er | CCR5 | 3A9 | BD | 556041 | 0.5 | ● |  |
| 167Er | CD16 | 3G8 | Miltenyi | 120-012-311 | 2 | ● |  |
| 168Er | CD11c | 3.9 | Biolegend | 301639 | 1.5 | ● |  |
| 169Tm | CXCR4 | 12G5 | BD | 555971 | 0.75 | ● |  |
| 170Er | CD14 | M5E2 | BD | 555396 | 1 | ● |  |
| 171Yb | IL-8 | G265.8 | BD | 554717 | 0.5 |  | ● |
| 172Yb | CD23 | 9P25 | Beckman | IMBULK1 | 2.5 | ● |  |
| 173Yb | CD141 | 1A4 | Fluidigm | 3173002B | 1.5 | ● |  |
| 174Yb | CD20 | 2H7 | BD | 556631 | 1 | ● |  |
| 175Lu | CCR7 | G043H7 | Miltenyi | 120-014-229 | 1 | ● |  |
| 176Yb | CADM1 | 3 E1 | Clinisciences MBL | CM004-3 | 0.5 | ● |  |

**Supplementary Table 1. Antibody panel for mass cytometry.**
